# To Cure Sometimes, To Comfort Always, To Hurt The Least, To Harm Never

**DOI:** 10.4103/0973-1229.27598

**Published:** 2006

**Authors:** Ajai Singh, Shakuntala Singh

Medicine has to engage in battle on numerous fronts. To further scientific enquiry without giving up on the art of caring. To find more efficient methods of care, ameliorate disease and attendant suffering, without overriding human rights. To experiment, carry out clinical trials and refine processes of experimentation, without allowing human beings, especially from underprivileged sections, to be coerced, subtly or otherwise, and/or recruited without informed consent. To continue to further patient welfare without snubbing the corporate enterprise of medicine. To further this enterprise, as it holds great promise for scientific advancement, without encouraging its equally great potential for malevolence.

## Cure And Comfort

In all this, what does the man of medicine, and all the ancillaries connected with him, do?

It is wise to remember that we attempt to find cures. But this at present is possible only sometimes, and in few conditions. That, however, is no excuse to give up the good fight to find them. This is especially important if well-being is our ultimate goal in medicine. Unless we find cures, how will the person be really well? Till such time as this happens, medicine can at least engage itself spiritedly in the three Ds- reduce distress, minimize disability, and prevent death. It can also offer comfort by both the caring attitude of medical personnel, and more humane and distress amelioration oriented advances of the enterprise of medicine. (For, an *enterprise* it has become for sure, whether you like to accept it that way or not.)

In other words, to cure *sometimes*; but to care for, and comfort, *always*.

## Hurt And Harm

There is a further thought which must engage our attention here. Medicine today holds immense potential to hurt and harm. Corporatisation of medicine can cause this as much as inefficiency of caregivers, dehumanization due to automation of medical care, as also changing social values, wherein the main yardstick of success (even of a medical man) is a balance sheet, and where it is old fashioned to think of medicine as a noble profession.

Hurt is inevitable in medical procedures. Even an injection hurts. A surgical incision does that as well. All medicines can hurt because of their side effects. Even psychotherapy can hurt, as it disturbs stable-unstable equilibriums, ostensibly to usher in better ones.

Hurt is integral to change. In society, in individuals. In disease. Harm, however, is not.

The difference between the two must be clarified. While both hurt and harm involve distress to the other, harm also involves malevolence in the perpetrator. The intention is the culprit. Let us explain with a few examples.

Hurt occurs when surgery is carried out. Harm occurs when it is carried out to keep a hospital running. Hurt occurs when a kidney is transplanted. Harm occurs when it is surreptitiously removed from a poor, unsuspecting, gullible patient. Hurt occurs when properly indicated drugs/procedures cause severe side effects. Harm occurs when drugs/procedures are prescribed/carried out knowing they have great chance of causing side effects in a vulnerable individual, and then heroically treating him, all the time prolonging hospital stay and inflating bills. Hurt occurs when tubes are inserted into the different natural orifices. Harm occurs when it is done to make a sickness appear serious, often creating new orifices, and prolonging hospital stay. Hurt occurs when a patient has to pay for a costly procedure, or when a patient has to pay huge hospital stay bills. Harm occurs when the costly procedure/hospital stay is carried out even if unwarranted, to recover running costs of an establishment.

*Hurt* occurs often while we effect cures and offer care. *Harm* occurs when manipulation and exploitation is perpetrated by caregivers, when unnecessary procedures are carried out, when bills are unduly inflated, when commerce supersedes care in a medical setup.

## The Bottom Line

Every procedure in medicine hurts. The intention should be to hurt the least, and carry out all steps in care and research to see this happens. Developing less distressing modes of therapy is a welcome step in this direction.

But what one must always ensure is that harm is never on the agenda.

In other words, what the medical enterprise of today must guarantee is to hurt the *least*, but to harm *never*.

This is all the more necessary as the present environment is thick with man's urges to prosper and enjoy personal fortune, even while the other's welfare is trampled upon. And what applies to society applies, unfortunately, also to its caregivers, even the medical man. He has to be especially careful he does not fall prey to this ever-present danger in his newfound prosperity of recent times.

Medicine means nothing if not this: to cure sometimes, to comfort always, to hurt little, to harm never.

